# A novel frameshift *GRN* mutation results in frontotemporal lobar degeneration with a distinct clinical phenotype in two siblings: case report and literature review

**DOI:** 10.1186/s12883-017-0959-2

**Published:** 2017-09-15

**Authors:** Takashi Hosaka, Kazuhiro Ishii, Takeshi Miura, Naomi Mezaki, Kensaku Kasuga, Takeshi Ikeuchi, Akira Tamaoka

**Affiliations:** 1 Department of the Neurology, Division of Clinical Medicine, Faculty of Medicine, University of Tsukuba, 1-1-1 Ten’noudai, Tsukuba, Ibaraki 305-8575 Japan; 2 Department of Molecular Genetics, Brain Research Institute, Niigata University, 1-757 Asahimachi, Niigata, 951-8585 Japan; 3 Department of Neurology, Brain Research Institute, Niigata University, 1-757 Asahimachi, Niigata, 951-8585 Japan

**Keywords:** Progranulin, Primary progressive aphasia, Corticobasal syndrome, Frontotemporal lobar degeneration, Phenotypic heterogeneity, Case report

## Abstract

**Background:**

Progranulin gene (*GRN*) mutations are major causes of frontotemporal lobar degeneration. To date, 68 pathogenic *GRN* mutations have been identified. However, very few of these mutations have been reported in Asians. Moreover, some *GRN* mutations manifest with familial phenotypic heterogeneity. Here, we present a novel *GRN* mutation resulting in frontotemporal lobar degeneration with a distinct clinical phenotype, and we review reports of *GRN* mutations associated with familial phenotypic heterogeneity.

**Case presentation:**

We describe the case of a 74-year-old woman with left frontotemporal lobe atrophy who presented with progressive anarthria and non-fluent aphasia. Her brother had been diagnosed with corticobasal syndrome (CBS) with right-hand limb-kinetic apraxia, aphasia, and a similar pattern of brain atrophy. Laboratory blood examinations did not reveal abnormalities that could have caused cognitive dysfunction. In the cerebrospinal fluid, cell counts and protein concentrations were within normal ranges, and concentrations of tau protein and phosphorylated tau protein were also normal. Since similar familial cases due to mutation of *GRN* and microtubule-associated protein tau gene (*MAPT*) were reported, we performed genetic analysis. No pathological mutations of MAPT were identified, but we identified a novel *GRN* frameshift mutation (c.1118_1119delCCinsG: p.Pro373ArgX37) that resulted in progranulin haploinsufficiency.

**Conclusion:**

This is the first report of a *GRN* mutation associated with familial phenotypic heterogeneity in Japan. Literature review of *GRN* mutations associated with familial phenotypic heterogeneity revealed no tendency of mutation sites. The role of progranulin has been reported in this and other neurodegenerative diseases, and the analysis of *GRN* mutations may lead to the discovery of a new therapeutic target.

## Background

Frontotemporal lobar degeneration (FTLD) is characterized by degeneration of the frontal and temporal lobes, and presents as a clinically heterogeneous disease. The pathological classification of FTLD is based on the molecular features of the disease-associated inclusion-forming proteins: FTLD-tau, FTLD-TDP, FTLD-FUS, and FTLD-UPS. Clinically, FTLD is classified into two subsets: behavioral variant FTLD (bvFTLD) and primary progressive aphasia (PPA), the latter of which includes semantic dementia and progressive non-fluent aphasia. In addition, FTLD can be concomitant with corticobasal degeneration (CBD), progressive supranuclear palsy (PSP), and motor neuron disease (MND) [1].

Progranulin is widely expressed in the central nervous system and is involved in immunomodulation as well as cell growth and proliferation. Since the first demonstration of FTLD-associated progranulin gene (*GRN*) mutation in 2006 [2, 3], more than 150 *GRN* mutations have been identified, including 68 pathogenic mutations. FTLD due to a *GRN* mutation is histopathologically characterized by ubiquitin-positive and TDP-43-positive inclusion bodies. While the most frequent clinical phenotype is bvFTLD, PPA and corticobasal syndrome (CBS) have also been reported [4–6]. There are also reports of clinical heterogeneity within a family [7, 8]. In addition, FTLD due to a *GRN* mutation is rare in Asian individuals, with an incidence of < 1% in Asians compared to an incidence of 5–10% in Europeans [9, 10].

In this report, we present the case of a 74-year**-**old Japanese woman with left-side atrophy in the frontal and temporal lobes and symptoms of progressive anarthria and non**-**fluent aphasia. We identified the cause to be a novel frameshift mutation in *GRN* that caused progranulin haploinsufficiency.

## Case presentation

**Fig. 1:**
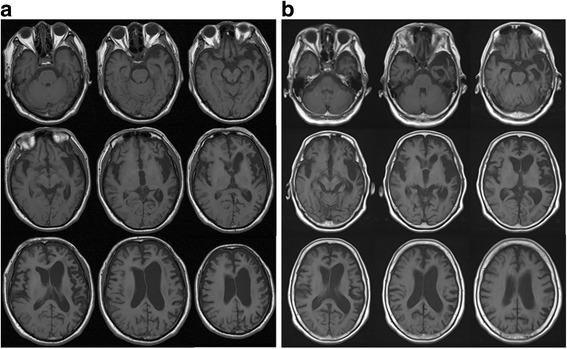
Brain MRI (axial T1-weighted images) of the patient’s brother (**a**) and the patient (**b**). **a** T1-weighted brain images of the patient’s brother at 4 years after disease onset. Atrophy was predominantly observed in the left hemisphere affecting the frontotemporal lobes. **b** T1-weighted brain images of the patient at 1 year after disease onset. Similar to her brother, atrophy was predominantly observed in the left hemisphere affecting the frontal and temporal lobes

A 74-year-old woman was referred to our hospital and admitted for progressive speech and language difficulties. The patient was unable to recall the names of things or persons and was unable to communicate with others for about 1 year prior to admission, though she was able to shop and do housework without difficulty. She had no significant medical history; however, regarding her family history, her elder brother had developed word-finding difficulty with verbal paraphasia and right-hand limb-kinetic apraxia at the age of 62 years of age, and was diagnosed with CBS at 69 years of age. He had frontal lobe signs such as forced grasping, total aphasia, and right-limb kinetic apraxia; moreover, brain magnetic resonance imaging (MRI) demonstrated frontal and temporal lobar atrophy dominantly affecting the left side (Fig. 1a). The patient’s brother and parents had passed away; therefore, we could not obtain their detailed clinical information.

Neurological findings indicated that our patient was lucid, but showed thought laziness. The cranial nerves, including those related to eye movement, were normal. The patient had normal muscle tonus and did not show muscle weakness or involuntary movement, but all extremity tendon reflexes were slightly increased. There was no evidence of sensory impairment or cerebellar ataxia. It was noted that speech required significant effort, was slow and non-fluent, and showed anarthria and aphasia. The patient’s Mini-Mental Scale Examination score was 4/30.

Language function was assessed using the Western Aphasia Battery (WAB) Japanese edition once and SLTA (standard language test of aphasia) two times within 2 months. The scores of WAB subtests were as follows: spontaneous speech, 13 points; auditory verbal comprehension, 5.5 points; repetition, 0 points; naming, 0 points; reading, 4.3 points; writing, 2.2 points; praxis, 6.8 points; and construction, drawing, block design & calculation, 6.6 points. Raven’s score was 25/37 (average ± standard deviation: 26.9 ± 5.4). Aphasia quotient was 36.8. The results of SLTA were similar to those of WAB. Naming, writing, and repetition were impaired. However, auditory verbal comprehension and reading concerning words and short sentences were relatively preserved. Spatial perception and visual perception were also normal. Verbal comprehension via visual perception was approximately normal. Therefore, it is likely that auditory verbal comprehension was complemented by visual perception. Constructional dysfunction, limb-kinetic apraxia, ideational apraxia, and motor apraxia were not observed. Laboratory blood examinations did not reveal any particular abnormalities that could have caused cognitive dysfunction. Cell counts and protein concentrations in the patient’s cerebrospinal fluid were within normal ranges, and concentrations of tau protein (282 pg/mL) and phosphorylated tau protein (31.3 pg/mL or lower) were also normal. Brain MRI demonstrated cerebral atrophy dominantly affecting the left frontotemporal lobes (Fig. 1b).

Clinically, the main patient symptoms were difficulty in verbal expression and non-fluent aphasia in the absence of visual memory impairment or behavioral abnormalities. On this premise, the patient was diagnosed with PPA according to Mesulam’s criteria [11]. Furthermore, the aphasia was classified as non-fluent progressive aphasia because, while speech itself required effort, the patient retained knowledge about objects and the ability to understand words. Brain MRI demonstrated cerebral cortical atrophy dominantly affecting the left frontal and temporal lobes, consistent with previous reports of non-fluent aphasia [4, 12]. Thus, FTLD was diagnosed according to the patient’s clinical symptoms. Since the patient’s elder brother had been diagnosed with CBS, and similar familial cases of FTLD due to *GRN* and microtubule-associated protein tau gene (*MAPT*) mutations had been reported [13], we performed genetic analyses on the patient.

Genomic deoxyribonucleic acid (DNA) was extracted from peripheral leukocytes isolated from the patient. The exon/intron boundary of *GRN* was amplified by polymerase chain reaction (PCR) according to a previously reported method [2] and the PCR products were sequenced in both directions. Briefly, blood was collected into a PAXgene® RNA tube, total ribonucleic acid (RNA) was extracted from the sample, and cDNA was prepared from total RNA by a reverse transcriptase reaction. cDNA was then amplified by reverse transcriptase–polymerase chain reaction (RT-PCR) (forward primer: 5′-ACCCAGGCTGTGTGCTG-3′; reverse primer: 5′-GACAGCCTCTGGGATTGGAC-3′) and the gene expression of *GRN* was analyzed. Then, the amplified PCR product was extracted and its sequence was analyzed.

**Fig. 2:**
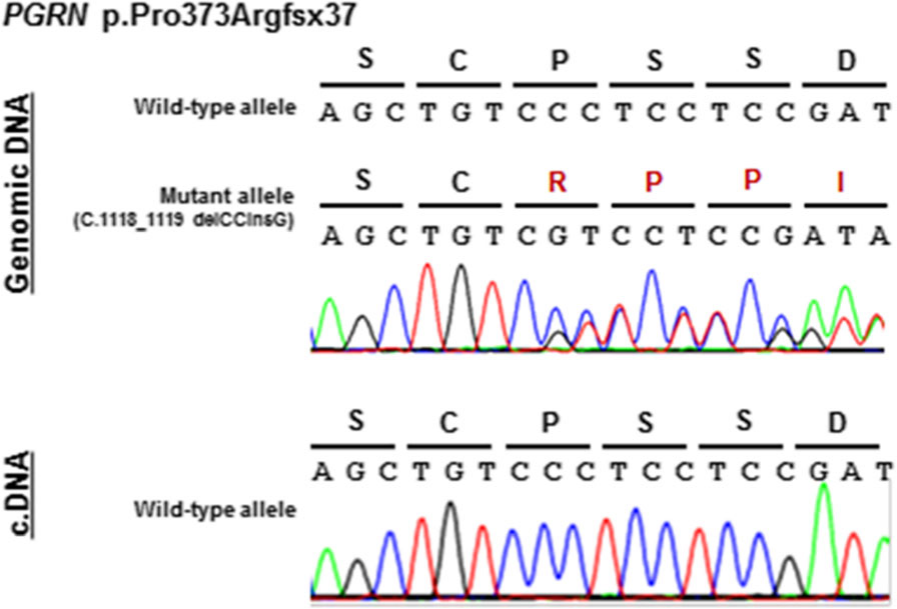
Genomic DNA and mRNA analyses. A sequential analysis of genomic DNA obtained from the patient revealed a novel mutation in *GRN* (c.1118_1119delCCinsG; p.Pro373ArgX38). RT-PCR analysis using cDNA prepared from the patient’s peripheral lymphocytes revealed no expression of the mutant allele, suggesting haploinsufficiency due to nonsense-mediated mRNA decay

The genetic examination identified a novel mutation (c.1118_1119delCCinsG) in exon10 of *GRN,* which was thought to cause a frameshift mutation (p.Pro373ArgX38). No pathological mutations of *MAPT* were identified*.* The *GRN* mRNA sequence was analyzed by RT-PCR; however, a mutant allele product was not detected, suggesting degradation of the mutant allele by the nonsense-mediated RNA decay system. Accordingly, haploinsufficiency due to reduced expression of progranulin was considered to be a possible pathogenic mechanism of FTLD in these cases (Fig. 2).

## Discussion and conclusions

Various types of mutations including aberrant splicing, gene deletion, frameshift, and nonsense mutations of *GRN* have been reported. These mutations are known to cause familial FTLD via progranulin haploinsufficiency [2, 3]. In the present case, our patient displayed PPA as a main symptom of progranulin haploinsufficiency due to a novel frameshift mutation of *GRN*. PPA, progressive difficulties with word recall and usage, and language comprehension impairments were apparent, whereas behavioral disinhibition, executive function, and memory impairments were not impaired in the early stages of disease (within 1 year after diagnosis). Other diseases known to cause PPA include FTLD, Alzheimer’s disease (AD), CBS, and Creutzfeldt–Jakob disease (CJD) [11]; however, a large number of studies reporting a link between *GRN* mutations and PPA suggest that *GRN* mutations should always be considered in the differential diagnosis of PPA. Similar symptoms, neuropsychological profile, and neuroimaging findings have been reported in a monozygotic twin pair with a *GRN* mutation [14]. In contrast, in our case, the patient’s brother presented distinct phenotypic characteristics (i.e., FTLD with PPA and CBS in the early stage). However, because the patient’s brother had already passed away, we could not obtain sufficient information to perform a genetic analysis. Table 1 provides a summary of known cases of *GRN* mutations that have been associated with familial phenotypic heterogeneity. The presence of familial phenotypic heterogeneity with respect to symptoms such as cognitive dysfunction and motor impairment has been reported in 17 families with *GRN* mutations [4–10, 12–19]. These studies reported significant variations in age of onset and mutation site, and motor neuron diseases were relatively uncommon. Families have also been reported with differing symptom laterality and different regions of brain atrophy. In a genetic analysis of 48 Japanese families with FTLD, PSP, or CBS [10], only one FTLD case with a *GRN* mutation was identified. Therefore, familial FTLD associated with *GRN* mutations is very rare. Furthermore, our report is the first to describe in detail distinct phenotypes within a family. Additional investigations of *GRN* mutations mediating different clinical phenotypes of neurodegeneration within a family are necessary.

**Table 1:** Familial cases presenting with distinct clinical phenotypes

Case	Age onset; number of patients	First symptom	Phenotype	Brain atrophy	Ethnic origin	*GRN* mutation
Rovelet-Lecrux et al., 2008 [15]	67,77; 2 patients	Language dysfunction	PPA	left > right	French	g.95_4390del
		Resting tremor	PD			
Spina et al., 2007 [13]	45,73; 2 patients	Involuntary arm movement	CBS	right > left	N/A	g.26C >A
		Cognitive decline	AD			
Beck et al., 2008 [4]	54–67; 10 patients	Language dysfunction	PPA	left > right (*n* = 2)	United Kingdom	g.90_91insCTGC
		Limb apraxia	CBS	right > left (*n* = 1)		
Skoglund et al., 2009 [12]	46–59; 10 patients	Language dysfunction	PPA	N/A	Swedish	g.102delC
		Limb apraxia	CBS			
Rademakers et al., 2007 [16]	62,66; 2 patients	N/A	FTLD, CBS	N/A	American	g.3240C > T
Masellis et al., 2006 [17]	57,62; 2 patients	Behavioral changes	FTLD	right > left	Canadian family of Chinese origin	g.1637G > A
		Axial and extremity rigidity	CBS			
Leverenz et al., 2007 [18]	35–69; 9 patients	Language dysfunction	FTLD	left > right (*n* = 3) right > left (n = 1)	American	g.1871A > G
		Anxiety, apathy	PPA			
		Parkinsonism	PD			
López de Munain et al., 2008 [19]	53,57; 2 patients	N/A	FTLD, CBS	N/A	Basque Country	g.1872G > A
	51,71; 2 patients	N/A	FTLD, CBS	N/A	Basque Country	g.1873G > A
	65; 2 patients	N/A	FTLD, CBS	N/A	Basque Country	g.1874G > A
	60; 2 patients	N/A	FTLD, CBS	N/A	Basque Country	g.1875G > A
	63–70; 4 patients	N/A	FTLD, CBS	N/A	Basque Country	g.1876G > A
	52; 2 patients	N/A	FTLD, ALS	N/A	Basque Country	g.1877G > A
Benussi et al., 2009 [5]	60–71; 5 patients	Language dysfunction	PPA	right > left	Italian	g.1977_1980delCACT
		Parkinsonism	CBS			
Kelley et al., 2009 [6]	N/A; 6 patients	N/A	FTLD, PD	symmetrical	American	g.2273_2274insTG
	N/A; 6 patients	N/A	FTLD, PD	right > left	American	g.2597delC
Pietroboni et al., 2011 [7]	47–79; 5 patients	Memory impairment, Acalculia	FTLD, AD	right > left (n = 1) symmetrical (n = 1) N/A (n = 3)	Italian	g.63_64insC
		Language impairment				
Rossi et al., 2011 [8]	47–80; 6 patients	Behavioural abnormality	FTLD Dementia	Left > right	Italian	g.1761_1762delCA
		Language dysfunction				
		Attention impairment				
The present case	75,62; 2 patients	Language dysfunction	PPA	left > right	Japanese	g.1118_1119delCCinsG
		Limb apraxia	CBS			

*AD* Alzheimer’s disease, *ALS* amyotrophic lateral sclerosis, *CBS* corticobasal syndrome, *FTLD* frontotemporal lobar degeneration, *GRN* progranulin gene, *N/A* not available, *PD* Parkinson’s disease, *PPA* primary progressive aphasia

As mentioned above, haploinsufficiency is thought to underlie the mechanism of *GRN* mutation-associated FTLD. Haploinsufficiency is a cause of autosomal genetic conditions when the protein expressed by a single allele is not sufficient to maintain its normal function (loss of function) [20]. On the other hand, in many autosomal dominant conditions, toxic gain of function or toxicity of excessive proteins are the cause of disease [21, 22]. In fact, an approximate 50% decrease in mRNA and 33% decrease in progranulin protein was reported in one *GRN* mutation carrier [1, 2]. It has thus been suggested that an effective therapeutic strategy would be to increase progranulin levels in patients [1]. The relationship between *GRN* genetic variability and the risk of developing a neurodegenerative disease such as AD or MND has been reported [1]. Yet, the exact functions of progranulin in the brain remain unclear, and its pathogenic involvement in neurodegenerative disorders is not known. Therefore, the accumulation of new cases of *GRN* mutations that display distinct clinical phenotypes within a family may be helpful not only for the elucidation of progranulin function, but also for the development of replacement therapies in FTLD and other neurodegenerative diseases due to *GRN* mutations.

## Abbreviations

AD: Alzheimer’s disease
ALS: Amyotrophic lateral sclerosis
bvFTLD: Behavioral variant frontotemporal lobar degeneration
CBD: Corticobasal degeneration
CBS: Corticobasal syndrome
CJD: Creutzfeldt–Jakob disease
DNA: Deoxyribonucleic acid
FTLD: Frontotemporal lobar degeneration
*GRN*: Progranulin gene
*MAPT*: Microtubule-associated protein tau gene
MND: Motor neuron disease
MRI: Magnetic resonance imaging
PCR: Polymerase chain reaction
PD: Parkinson’s disease
PPA: Primary progressive aphasia
PSP: Progressive supranuclear palsy
RNA: Ribonucleic acid
RT-PCR: Reverse transcriptase–polymerase chain reaction
